# Climate change, armed conflict, forced displacement, and epidemic-prone diseases: an exploratory study in northern Syria

**DOI:** 10.1186/s12889-025-23918-3

**Published:** 2025-08-04

**Authors:** Maia C. Tarnas, Naser Almhawish, Ruwan Ratnayake, Yasir Elferruh, Ibrahim Aladhan, MHD Bahaa Aldin Alhaffar, Aula Abbara

**Affiliations:** 1 Department of Population Health and Disease Prevention, University of California, Irvine, USA; 2 Syria Public Health Network, London, UK; 3 Department of Infectious Disease Epidemiology and International Health, London School of Hygiene and Tropical Medicine, London, UK; 4 Early Warning Alert and Response Network, Assistance Coordination Unit, Gaziantep, Türkiye; 5 Syrian Environmental Protection Agency, Gaziantep, Türkiye; 6 Department of Global Public Health, Karolinska Institute, Stockholm, Sweden; 7 European Centre for Disease Prevention and Control, Stockholm, Sweden; 8 Department of Infectious Diseases, Imperial College, St. Mary’s Hospital, London, UK

## Abstract

**Introduction:**

Northern Syria is particularly vulnerable to the joint effects of climate change and conflict. This has contributed to numerous infectious disease outbreaks which disproportionately affect people who have been forcibly displaced. We aimed to assess the associations between environmental factors, conflict, displacement, and two types of epidemic-prone diseases in northern Syria: suspected respiratory infections and diarrheal diseases.

**Methods:**

We used data from the Early Warning Alert and Response Network (EWARN) syndromic surveillance system between 2016 and 2023 on two suspected respiratory infections and five suspected diarrheal diseases. These cases were aggregated by disease type at the district-week level. For each disease type, we used a generalized additive model with a negative binomial probability distribution that accounted for several environmental variables (including precipitation, surface water, temperature, humidity, and vegetation), displacement, conflict events, total consultations, prior disease cases, seasonality, and spatial factors. Seasonal-trend decomposition with locally estimated scatterplot smoothing was also used to detect trends amidst seasonal fluctuations.

**Results:**

Over 21 districts in 5 governorates, 8,774,734 suspected respiratory infections and 6,903,396 suspected diarrheal disease cases were reported. Proportionate morbidity for both disease types began increasing in late 2018 and early 2019 with fluctuations; this varied by governorate. Scaled mean temperature (SD: 11.59°C) was associated with decreased risk of respiratory infections (IRR: 0.92; 0.87-0.98) but increased risk of suspected diarrheal disease (1.06; 1.03-1.09) in the same week and up to 8 weeks and 4 weeks later, respectively. Precipitation exhibited similar contrasting risk patterns. Surface water and vegetation levels also corresponded to changes in disease transmission risk. The interaction between high levels of displacement and conflict was associated with increased risk for both, though suspected diarrheal diseases had a lower threshold for increased risk.

**Conclusions:**

Conflict, environmental factors, forced displacement, and infectious diseases are inextricably linked in northern Syria. These findings can inform public health preparedness and anticipatory activities and policies that address the effects of climate change on infectious diseases. This is especially relevant as Syria enters a new geopolitical chapter following the fall of the Assad regime, with changing health needs, population movement, and new opportunities for health system recovery.

**Supplementary Information:**

The online version contains supplementary material available at 10.1186/s12889-025-23918-3.

## Introduction

Climate change is a major global threat bringing rising temperatures, heavy precipitation, and droughts (agricultural and ecological). These shocks drive increased infectious disease transmission [1]. Though the incidence of vector-borne diseases deserves special consideration due to vector-specific changes, diarrheal and respiratory infections are also vulnerable to changes in seasonal patterns and other climate factors [2–4]. Prior studies have established associations between environmental processes, climate change, and diarrheal and respiratory infections, though the magnitude of individual relationships (i.e., between specific environmental processes and diseases) are often context specific [2–4]. In areas affected by conflict, and especially those with large-scale forced displacement, climate change can compound existing vulnerabilities. These joint effects can include increased disease susceptibility among the population (through compromised immunity and malnutrition, for example), coincident environmental changes which favor infectious disease outbreaks, cessation of vaccination programs, the inability to detect and respond to outbreaks in a timely manner, breakdown of public health systems, and famine [5]. This is especially true in Syria, which faced more than 13 years of conflict, forced displacement of more than half of its pre-conflict population of 22 million, breakdown of water and sanitation systems, and fragmentation of its health system [6]. These factors have contributed to numerous outbreaks, including of polio, measles, COVID-19, leishmaniasis, cholera, and several vaccine-preventable diseases [6]. In northern Syria, natural disasters including the earthquakes of February 2023 have also contributed to the risk of outbreaks [7].

In addition to protracted conflict, Syria has also experienced severe droughts, floods, and extreme heat, with rural areas in the north particularly affected [8]. These have had reverberating effects on food security, agriculture, and human health [9]. Since 1990, Syria has seen significantly decreased rainfall, especially in the wet season [10, 11]. The wet season is important to Syria’s overall water availability, and this decline makes the country increasingly vulnerable to droughts. The severity and intensity of droughts have been increasing over the past 25 years, causing an estimated cumulative water loss of 2.2 billion cubic meters as of 2022 [12, 13]. Droughts are likely impacted by the region’s increasing temperatures, which are expected to warm between 3.5 °C and 7 °C by 2070 to 2099 [14]. The warming in this area has occurred primarily in the last 20 years and has largely outpaced the global mean temperature increase [15]. Increased mean temperature can both exacerbate the impact of other environmental events (including droughts through accelerating the rate of water evaporation) and have direct effects on human and animal health [16]. Humidity and vegetation levels are also closely linked to these processes. For example, warmer air can hold more moisture, leading to increased humidity and heavier precipitation. Vegetation levels likewise shape humidity and rainfall patterns, which can in turn influence the location and duration of flooding [17]. Human-induced over-extraction of groundwater has also compounded Syria’s vulnerability to adverse environmental events. Such environmental changes, though only discussed in broad strokes here, are likely a factor in the continuous and compounding daily risk for the 16.7 million people who require humanitarian aid, of whom 7 million are children and 4.5 million are women [9, 18]. Despite these factors, we note little academic exploration of the impact of climate change on the health of Syria’s population.

In December 2024, the Assad regime fell after half a century of dictatorship following a major offensive by opposition forces. As Syria’s transition moves forward, it is increasingly clear that the fractured healthcare system which evolved during the conflict - with subnational systems operating under independent governing structures - is neither desirable nor sustainable [19]. This fragmentation was also reflected in how infectious diseases were monitored throughout the conflict: in northern Syria, the Assistance Coordination Unit (ACU) established the Early Warning Alert and Response Network (EWARN) in 2013, existing alongside the Early Warning Alert and Response System (EWARS) that was set up in 2012 with the WHO to monitor areas under government control [20–22]. These systems are now transitioning to a country-wide unified approach [19]. The aim of this research is to explore how environmental factors, forced displacement, and conflict are associated with suspected respiratory infections and diarrheal disease in northern Syria between 2016 and 2023 using EWARN data, as their joint impacts have likely been contributors to disease trends. Though the geopolitical context has changed (and continues to change rapidly) since this study period, such findings may be useful in establishing priorities in disease prevention and climate change adaptation in Syria’s new chapter.

## Methods

**Fig. 1:**
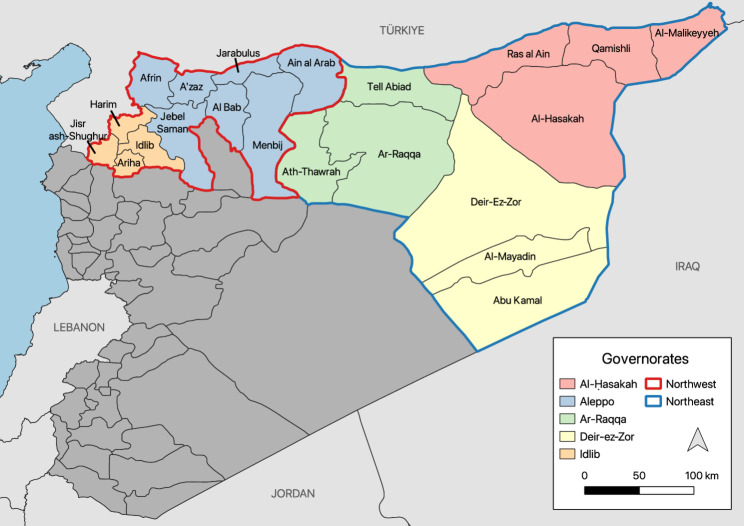
Map of study area. The study only included data from northern Syria because EWARN’s coverage is limited to that region

This is an ecological study focused on northern Syria between 2016 and 2023 (Fig. 1). As of November 2024 in northwest Syria, 4.24 million people require humanitarian assistance (82% of the region’s population), with roughly 3.54 million displaced internally, 2.0 million of whom live in camps [23]. Nearly 700,000 IDPs reside in northeast Syria, around 150,000 of whom live in camps [24, 25]. The largest camp, Al Hol, housed over 50,000 IDPs in Al-Hasakah governorate [25]. Both regions of northern Syria have been subject to intense violence by the former Syrian regime [26].

### Data sources

Weekly EWARN data from January 2016 to September 2023 (401 weeks) on suspected respiratory infections, suspected diarrheal disease, and the total number of clinical consultations was used. This disease surveillance system provides continuous data using syndromic case definitions for key epidemic-prone diseases (e.g., cholera, shigella dysentery, typhoid, influenza, etc.) and are intended to provide early warning of an emerging outbreak. ACU provided data at the district level on the following syndromes: influenza-like illness (ILI), severe acute respiratory infection (SARI), acute watery diarrhea (AWD, indicative of suspected cholera), acute bloody diarrhea (ABD, indicative of suspected shigella dysentery), acute jaundice syndrome (AJS, indicative of suspected hepatitis), suspected typhoid fever (STF), and AOD (acute diarrhea of other origin). For our analyses, suspected respiratory infections (SARI and ILI) and diarrheal disease (AWD, ABD, AJS, STF, and AOD) were aggregated to reflect the mode of transmission. EWARN follows WHO guidance on syndromic case definitions [27, 28]. To calculate proportionate morbidity ratios, the weekly sum by disease type was divided by the total number of consultations for each district-week. Districts that were missing more than half of the weeks of data were excluded in order to preserve the time series length and reduce potential bias.

Weekly data on precipitation, surface vegetation, surface water, surface temperature, and humidity were extracted using Google Earth Engine to create time series that may be predictive of disease incidence, given the established associations between environmental factors and disease dynamics. Daily precipitation data were extracted from the Climate Hazards Center InfraRed Precipitation with Station (CHIRPS) dataset at a 0.05° spatial resolution [29]. Daily mean precipitation for each district was calculated and then summed over the week to create weekly measurements. Normalized difference vegetation index (NDVI) values were pulled from the Terra MODIS satellite, available every 16 days at 250 m [30]. NDVI provides a measurement of surface vegetation on a scale of −1 to 1, with − 1 indicating extremely low surface vegetation levels and 1 indicating extremely high levels. MODIS red and shortwave infrared 2 bands, available every 8 days at a 500 m scale, were used to calculate a surface water index which ranges from − 1 to 1, with − 1 indicating extremely low surface water levels and 1 indicating extremely high levels [31]. We also used MODIS to extract daily mean, minimum, and maximum land surface temperature values at a 1 km scale [32]. Mean daytime and nighttime temperatures were averaged to find total mean temperature, and the absolute minimum and maximum values for each week were used to calculate a temperature range. Lastly, monthly humidity mean and range were extracted from the Famine Early Warning Systems Network (FEWS NET) Land Data Assimilation System at 0.1° spatial resolution [33]. All environmental variables other than precipitation and temperature were converted to weekly intervals using weighted means, and all data were mean-centered and standardized except for NDVI and humidity range, which were log-transformed due to skewness. Weeks with missing and zero values could be differentiated, and we did not perform interpolation as missingness was minimal. Changes in environmental variables over the study period and between seasons (wet and dry) were analyzed using two sample t-tests.

Likewise, population movement and conflict events were thought to together create optimal environments for disease transmission and potentially increase contacts between susceptible and infected persons and hence, predict disease incidence. Governorate-level monthly displacement data describing population changes were sourced from Humanitarian Data Exchange [34]. To transform these into district-week intervals, total governorate-level monthly displacement was divided evenly between all districts in the governorate and across the weeks in each month. Lastly, violent conflict events were pulled from the Uppsala Conflict Data Program and were aggregated at the district-week level [35]. Events that spanned multiple days were assigned to the week in which the event began. We also extracted the geometric centroids by using QGIS (v3.22.10) to capture baseline spatial factors not accounted for by other variables.

### Seasonal-trend decomposition

To assess the underlying seasonal trends for each disease type, we used seasonal-trend decomposition with locally estimated scatterplot smoothing (LOESS) on each proportionate morbidity time series. This method splits the time series into three components: (1) seasonal, (2) trend, and (3) remaining random variation.

### Model building

We used generalized additive models (GAM) with a negative binomial probability distribution to account for overdispersion in the case data and to more easily interpret the potentially non-linear contributions of each potential predictor on the disease outcomes. Suspected respiratory infections and diarrheal diseases were modeled separately. Given that population-level impacts of environmental effects on transmission are delayed, weekly lags of 1 to 8 weeks were tested for each environmental variable to account for any delayed effects [36–38]. We conservatively set the maximum lag as approximately 10 times (rounded to the nearest week) the maximum incubation period of common acute respiratory viral infections and viral gastroenteritides (i.e., estimated by meta-analysis to be approximately 5 days at the highest end of the range) to cover multiple generations of infections [39, 40]. In instances where the lagged terms exhibited high concurvity when modeled as splines (indicating that the smoothed term could be approximated by others), we excluded weeks necessary to address this, resulting in 0–8-week lags for surface water and temperature range, 0-, 4-, and 8-week lags for mean temperature, mean humidity, and humidity range, and 0- and 8-week lags for NDVI. We also tested several temporal aggregations of weekly precipitation in each model, given the established potential of cumulative rainfall (or lack thereof) to influence disease dynamics [41]. We included a 4-week (i.e., the total rainfall in one week and the three prior weeks) and 6-week aggregation in the suspected respiratory infection and diarrheal disease models, respectively, based on model fit and scientific hypotheses. These aggregations were categorized using quartiles to preserve their nonlinear nature while increasing interpretability. The 4-week aggregations were low (0.00–0.36 mm), intermediate (0.37–14.67 mm), medium (14.68–36.35 mm), and high (36.36–254.28 mm), and the 6-week aggregations were low (0.00–1.81 mm), intermediate (1.82–25.11 mm), medium (25.12–53.55 mm), and high (53.56–328.92 mm).

We included displacement and conflict events as an interaction term; both were tested as individual variables in the model but were ultimately specified as an interaction based on model fit and scientific hypotheses (i.e., conflict events spur displacement, and vice versa) (Additional file 1). Two temporal variables were included in the final model: one accounted for continuous time during the study period and the other for seasonality. We also controlled for the total number of weekly consultations in the model and included an autoregressive term to account for the number of suspected disease cases in the prior week. All analyses were run in R version 4.0.4.

## Results

### Descriptive results

**Fig. 2:**
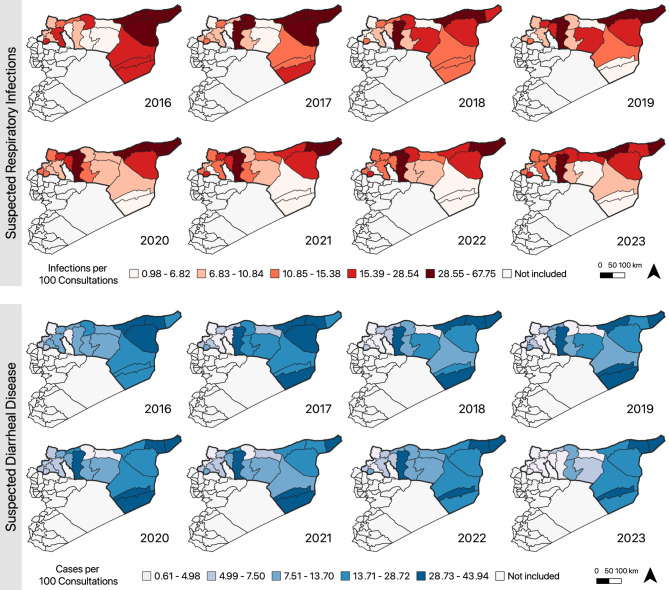
Map of annual suspected respiratory infections and suspected diarrheal disease cases per 100 consultations. Data were extracted from the EWARN syndromic surveillance system, which only covers northern Syria

A total of 8,774,734 suspected respiratory infections and 6,903,396 suspected diarrheal disease cases were reported from January 2016 to September 2023 in 21 districts. ILI comprised the majority of suspected respiratory infections (*n* = 8,616,351; 98.2%). Suspected respiratory infections were highest in Harim (*n* = 1,199,516; 13.7%), A’zaz (*n* = 920,207; 10.5%), and Jebel Saman districts (*n* = 692,107; 7.9%) (Fig. 2). However, Menbij (35.9 cases per 100 consultations), Al-Malikeyyeh (33.3 per 100), and Qamishli (31.8 per 100) had the highest proportionate morbidity for suspected respiratory infections over the study period.

Suspected diarrhea cases consisted primarily of AOD (*n* = 5,579,360; 80.8%), AWD (*n* = 639,749; 9.3%), and STF (*n* = 329,808; 4.8%). AWD cases surged in 2022 which reflected the cholera outbreak in the region; a total of 15 AWD cases occurred between 2016 and 2021, whereas 108,362 and 531,372 were reported in 2022 and 2023, respectively. Most diarrhea cases occurred in Deir-ez-Zor (*n* = 875,203; 12.7%), Harim (*n* = 774,428; 11.2%), and Qamishli (*n* = 531,501; 7.7%), though Menbij (33.0 per 100), Abu Kamal (32.8 per 100), Al-Malikeyyeh (31.4 per 100), and Qamishli (29.0 per 100) had the highest proportionate morbidity (Fig. 2).

**Fig. 3:**
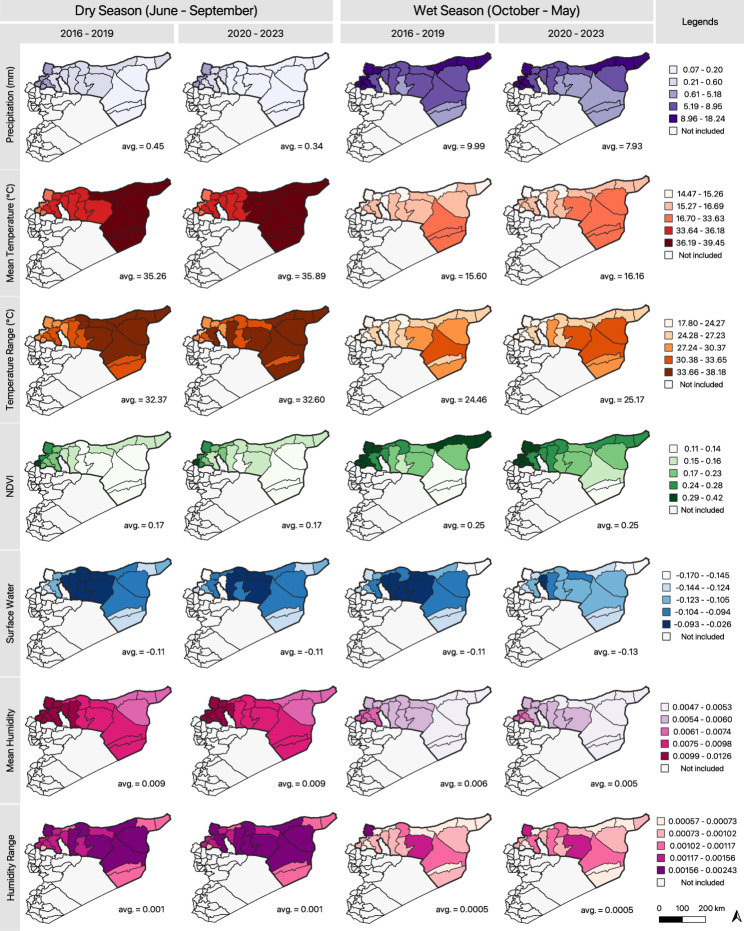
Map of district-level means for each environmental variable between 2016–2019 and 2020–2023 in the dry and wet seasons. Values to the right of each map are the total mean for all included districts for each period

Environmental factors varied seasonally and over the study period (Fig. 3). Mean weekly precipitation declined by 1.43 mm between 2016 and 2019 and 2020–2023 (*p* < 0.0001), and wet seasons across the study period experienced 8.61 mm more rainfall than dry seasons (*p* < 0.0001). After accounting for seasonality, weekly rainfall peaked in December 2018, following a steady increase. Mean weekly surface water subsequently had a peak in March 2019 (also after a steady increase), but overall did decline between 2016 and 2019 and 2022–2023 (*p* < 0.0001).

Between 2016 and 2019 and 2020–2023, mean weekly temperature increased by 0.65 °C (*p* = 0.01). Temperatures were also 19.69 °C warmer in the dry season (min: 24.18 °C, max: 44.20 °C, IQR: 5.32 °C) than in the wet season (min: −4.44 °C, max: 36.8 °C, IQR: 13.20 °C). Temperature ranges in the 2020–2023 period were wider (*p* = 0.0004) than those in the 2016–2019 period. Lastly, mean humidity was significantly higher during the dry season than in the wet season (*p* < 0.0001) but did not significantly change over the study period (*p* = 0.146) (Fig. 3).

### Seasonality

**Fig. 4:**
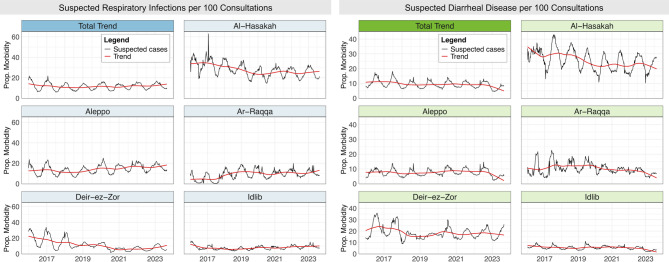
Trends in suspected respiratory infections and diarrheal disease by governorate. Black lines show the weekly proportionate morbidity and red lines show the trends alone after removing the seasonal and random variation components through seasonal-trend decomposition with LOESS

Following seasonal-trend decomposition with LOESS, proportionate morbidity for suspected respiratory infections and diarrheal disease decreased at the beginning of the study period and began to increase in late 2018 and early 2019 with subsequent fluctuations (Fig. 4). However, these trends were not consistent across governorates. Suspected respiratory infection proportionate morbidity consistently declined in Al-Hasakah, Deir-ez-Zor, and Idlib governorates until 2020, 2021, and 2018, respectively. In Aleppo and Ar-Raqqa, however, it increased relatively consistently with fluctuations throughout. Trends in suspected diarrheal disease proportionate morbidity across governorates were more nuanced. Deir-Ez-Zor and Aleppo experienced relatively stable increasing trends over several years throughout the study period, whereas the opposite was true in Ar-Raqqa and Idlib. Ar-Raqqa had a notable period of elevated cases between late 2017 and late 2019, and all governorates experienced a declining trend at the end of the study period. We note that case definitions changed at the onset of the AWD outbreak in 2022 which led to far fewer AOD cases reported, and the concurrent declining trend likely reflects this.

### Model results

**Fig. 5:**
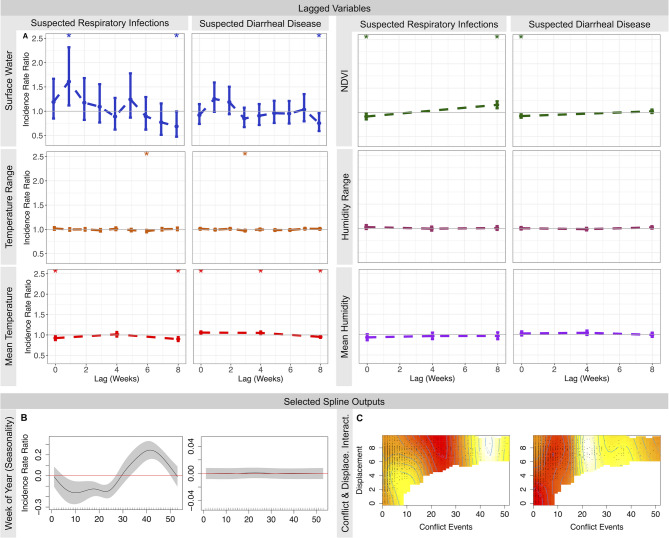
Model outputs for lagged environmental variables in each model to show changing associations over time (**A**). Significant weeks are marked with an asterisk. The bottom of the figure shows select spline outputs from each model. The seasonality splines (**B**) are considered significant when the black line (IRR) and grey shaded areas (95% CIs) are all either above or below the red line. In the conflict and displacement interaction (**C**), reds indicate lower risk and yellows indicate higher risk. The remaining spline outputs can be found in Additional file 2

Significant environmental variables overlapped between both disease types (Table 1; Fig. 5A). The spline outputs can be found in Additional file 2. Scaled mean temperature (SD: 11.59 °C) was associated with decreased risk of suspected respiratory infections in the same week (IRR: 0.92; 95% CI: 0.87–0.98) and up to 8 weeks later but increased risk of suspected diarrheal disease in the same week (1.06; 1.03–1.09) and 4 weeks later. Higher NDVI values in the same week, indicating increased vegetation levels, were associated with decreased risk for both disease types. This changed to a positive association after 8 weeks for suspected respiratory infections. High levels of precipitation were associated with reduced risk of suspected respiratory infections (0.92; 0.87–0.98) when compared to low precipitation levels, and increased surface water was associated with higher risk of disease in the following week (1.61; 1.12–2.32). All levels of precipitation corresponded to increased diarrhea risk.

**Table 1:** Model outputs for suspected respiratory infection and suspected diarrheal disease GAMs

Covariate	Suspected Respiratory Infections		Suspected Diarrheal Disease	
	IRR (95% CI)	*p*-value	IRR (95% CI)	*p*-value
Precipitation^†^				
Low	Comparison		Comparison	
Intermediate	0.96 (0.92–1.00)	0.063	1.05 (1.02–1.08)	0.0001
Medium	0.95 (0.90–1.01)	0.088	1.08 (1.04–1.11)	< 0.0001
High	0.92 (0.87–0.98)	0.014	1.07 (1.03–1.12)	0.0003
Surface water				
No lag	1.19 (0.85–1.67)	0.313	0.92 (0.74–1.15)	0.457
1-week lag	1.61 (1.12–2.32)	0.010	1.25 (0.99–1.59)	0.064
2-week lag	1.18 (0.82–1.68)	0.376	1.19 (0.94–1.50)	0.152
3-week lag	1.09 (0.77–1.56)	0.623	0.85 (0.67–1.07)	0.170
4-week lag	0.89 (0.62–1.27)	0.523	0.91 (0.72–1.15)	0.414
5-week lag	1.24 (0.87–1.78)	0.235	0.96 (0.76–1.21)	0.733
6-week lag	0.90 (0.62–1.29)	0.554	0.95 (0.75–1.21)	0.681
7-week lag	0.77 (0.51–1.16)	0.212	1.03 (0.79–1.35)	0.801
8-week lag	0.69 (0.47–0.99)	0.046	0.75 (0.59–0.96)	0.022
Temperature (mean)				
No lag	0.92 (0.87–0.98)	0.005	1.06 (1.03–1.09)	< 0.0001
4-week lag	1.01 (0.96–1.07)	0.601	1.05 (1.02–1.09)	0.003
8-week lag	0.90 (0.85–0.95)	< 0.0001	0.95 (0.93–0.98)	0.0004
Temperature (range)				
No lag	1.02 (0.99–1.05)	0.187	1.01 (1.00–1.03)	0.161
1-week lag	1.00 (0.97–1.02)	0.883	0.99 (0.98–1.01)	0.549
2-week lag	1.00 (0.98–1.03)	0.938	1.01 (1.00–1.03)	0.165
3-week lag	0.98 (0.96–1.01)	0.150	0.98 (0.96–0.99)	0.003
4-week lag	1.01 (0.98–1.04)	0.388	1.00 (0.98–1.02)	0.933
5-week lag	0.99 (0.96–1.01)	0.275	0.99 (0.97–1.00)	0.113
6-week lag	0.96 (0.94–0.99)	0.006	0.99 (0.97–1.00)	0.129
7-week lag	1.00 (0.98–1.03)	0.785	1.01 (1.00–1.03)	0.094
8-week lag	1.01 (0.98–1.04)	0.468	1.01 (0.99–1.03)	0.222
Humidity (mean)				
No lag	0.93 (0.86–1.01)	0.088	1.03 (0.98–1.08)	0.250
4-week lag	0.97 (0.89–1.05)	0.409	1.04 (0.99–1.10)	0.092
8-week lag	0.97 (0.89–1.06)	0.464	0.99 (0.94–1.05)	0.842
Humidity (range)				
No lag	1.03 (0.99–1.07)	0.186	1.00 (0.98–1.03)	0.832
4-week lag	0.99 (0.95–1.04)	0.779	0.98 (0.96–1.01)	0.253
8-week lag	1.01 (0.97–1.05)	0.718	1.02 (1.00–1.04)	0.117
NDVI				
No lag	0.91 (0.86–0.98)	0.009	0.92 (0.89–0.96)	< 0.0001
8-week lag	1.15 (1.08–1.23)	< 0.0001	1.02 (0.99–1.06)	0.230

† Precipitation was aggregated at 4 weeks for suspected respiratory infections and 6 weeks for suspected diarrheal disease

The interaction between displacement and conflict was associated with heightened risk of both suspected respiratory infections and diarrheal disease at certain levels (Fig. 5C). Increased risk of suspected respiratory infections was strongest with high levels of conflict (approximately > 35 conflict events) and displacement above 150 individuals in the same week (mean displacement for the study period was around 430 individuals per week). There was also increased risk, though to a lesser effect, for lower levels of conflict (around 5–15 events) and displacement. The greatest increased risk for suspected diarrheal disease occurred at lower levels of conflict and displacement compared to suspected respiratory infections (> 20 conflict events and > 50 displaced individuals in the same week). There was also significant risk with high displacement but low conflict, though this was marginal.

## Discussion

Conflict, environmental factors, and disease incidence are increasingly intertwined. Our study finds that several of the environmental factors evaluated were associated with changes in suspected diarrheal disease and respiratory infections in northern Syria, but in line with known biology, these associations were not often consistent between the two disease types. We also note that the combination of high levels of displacement and conflict was consistently associated with increased risk of both suspected diarrheal disease and respiratory infections, which accords with existing literature [42]. Syria has already experienced severe repercussions of climate change on its environment (e.g., through droughts, floods, and extreme heat), and this will likely continue in the future. We note that northeast Syria is particularly affected with higher temperatures in both the dry and wet seasons, less surface vegetation, and less surface water than northwest Syria; it is also more affected by interruptions of its key water stations, particularly Allouk water station. This may partially explain the higher proportionate morbidity, especially for suspected diarrheal disease, in northeastern governorates. Understanding the relationships between these environmental factors and infectious diseases, while also incorporating relevant conflict-related factors, is critical to prepare for changing disease burdens resulting from shifting environments. It can also help support regional and seasonal mitigation strategies given the localized differences seen in environmental factors and the burden of infectious diseases.

Mean temperature, precipitation, surface water, and vegetation levels are particularly relevant in the Syrian context. We found that increasing mean temperatures were associated with decreased risk of suspected respiratory infections but increased risk of suspected diarrheal disease across a lag period extending to 8 and 4 weeks respectively, which accords with prior work. There is a plausible biological-environmental mechanism for these relationships wherein, for instance, lower temperatures cause lower humidity and conditions facilitating respiratory transmission [43]. Warming temperatures in Syria have already largely outpaced global mean temperature increases (with no indication of cessation), indicating that risk of suspected diarrheal diseases may likewise increase [14, 15]. However, decreasing precipitation levels, which Syria has experienced since 1990, may increase suspected respiratory infection risk while decreasing risk of suspected diarrheal disease [10, 11]. We also found that vegetation levels were associated with reduced suspected respiratory infection risk in the same week but increased risk after 8 weeks. There are several possible explanations for this. Higher vegetation levels may create, over several weeks, environments that are more hospitable to disease spread. This could be through multiple pathways, including interactions with other environmental factors, increasing exposure to irritants (e.g., mold and pollen) that are risk factors for respiratory infections, or changing individual-level patterns, gradually increasing exposures and impacting health behaviors. The findings may also reflect different seasonality between NDVI and suspected respiratory infections, whereby their respective seasonal peaks occur at different times. While we account for seasonality with a week-of-year spline, there may be additional seasonality components that are meaningful to this relationship.

In Syria, as in elsewhere, it is important to note that changes in environmental factors are neither unidimensional nor consistent across time and space. While Syria is generally receiving less rainfall than before, there have been several instances of heavy rainfall (which likely also impacts vegetation levels) with widespread flooding, particularly affecting northwest Syria [44]. Such flooding may acutely increase the risk of suspected respiratory infections in the subsequent weeks, as pooled waters may harbor pathogens and other contaminants deleterious for respiratory health [45]. Flooding may also increase displacement and housing overcrowding in many contexts, which is an ideal context for respiratory transmission. This illustrates that when considering the potential effects of environmental events on disease incidence, long-term mean changes and as well as short-term climatic events must both be considered, as must unique spatial and temporal contexts. Likewise, it also highlights that certain diseases can be expected to increase by mode of transmission, according to changes in climate variables.

We also found the interaction between conflict and climate to be significant for both disease types. Conflict has been linked to increases in infectious disease risk throughout the literature, including in Yemen and Sudan [46, 47]. Though we tested each model with conflict and displacement modeled independently, we found that the interaction term produced a stronger model fit and is more consistent with the reality in northern Syria of intense conflict followed by mass displacement during the study period. Increased risk of suspected diarrheal disease at lower levels of conflict and displacement may be triggered by single but large-impact conflict events relating to destruction of water-related infrastructure, which could have a rapid effect on both displacement and waterborne disease cases. Allouk water station in Al-Hasakah is one example of this. The water station serves over 460,000 individuals in northeast Syria yet has faced recurrent direct attacks, discriminatory operation by the Turkish government (e.g., the station was cut off for five days in 2020 without reason), and its use in bargaining. This politicalization impeded the response to the 2022–2023 cholera outbreak in the region as regions downstream of Allouk were unable to access adequate water [48–50].

Two strengths of the study include biologically plausible findings and high explanatory power of the variables used. However, this study does have its limitations. The surveillance data used captures suspected but not laboratory-confirmed cases of disease amongst those persons who have access to health care, and is therefore biased against the specificity of case definitions in favor of the sensitivity of disease detection. In this exploratory analysis, we considered environmental variables and their lags individually in our model and did not consider any potential interplay between them. Future work should consider a conceptual causal model in evaluating linkages. The environmental variables may represent similar impacts on disease incidence due to their linkages in the causal chain. For instance, increased humidity may be a consequence of increased precipitation and exhibit a single effect. Each model was initially run with all environmental variables and their lags specified non-parametrically, though few exhibited non-linear behavior and thus we opted to specify them linearly to increase the models’ interpretability.

This study also uses a coarse measure of displacement, based on the available governorate-level data, that required several assumptions to move from a monthly governorate-level measurement to a weekly district-level. We evenly distributed the total reported monthly displacement for each governorate across each week and district within that month and governorate; this loses any variation between weeks or between the districts within a given governorate that may have affected incidence. Such spatial and temporal smoothing may obscure differences relevant to disease transmission and thus bias our results. However, it reflects our desire to capture displacement levels relative to districts over a multiyear time series, and we anticipate that this did not severely impact our results. However, future work that looks at these relationships (across environmental factors, conflict, and displacement) at smaller spatial scales would be important should those data become more readily accessible. Lastly, there may be other relevant variables within the causal chain, such as socioeconomic factors and conditions of displacement settlements, that would likely impact disease dynamics but that we were unable to capture.

The December 2024 fall of the Assad regime and subsequent establishment of a transitional government has undoubtedly moved Syria into a new era. Crucial to this transition will be the creation of robust and resilient healthcare and surveillance systems that can respond to the complex health needs of a population subjected to over a decade of violent conflict. This also presents a rare opportunity to plan for Syria’s future, which can include emerging strategies relating to climate change and disease prevention. Raleigh et al. (2024) have explored the possibility of climate change adaptation interventions relating to natural resource management, climate-smart agriculture, and drought-control measures, underpinned by community-led initiatives and local governance that could be appropriate for fragile, conflict and violence-affected countries [51]. In Syria, this can be informed by research that focuses on evaluating the forecasting capacity of environmental prediction variables, permitting anticipatory action for epidemic prevention according to long-term changes in climate variables and acute climate-related events [52].

Preparedness efforts that focus on climate change adaptation, with an emphasis on those relating to precipitation and temperature changes, will be important in this region. Syria currently does not have a formal climate change adaptation strategy [53], and the implementation of any future adaptation measures would require a revitalization of international financial support for the region [54]. Increased infectious disease risks are only one of several expected deleterious outcomes of these interplays. Malnutrition, livelihood generation, and the availability of habitable land have already been impacted by climate change and will continue to be so; each of these will also have direct impacts on individuals’ disease susceptibility. Though integrating climate considerations amidst ongoing violent conflict - and now a transitioning government - is certainly not ideal, it is necessary [55]. Such actions, in conjunction with ongoing advocacy and accountability efforts related to conflict, can work toward reducing the suffering experienced by Syrians, many of whom have only known these conditions.

## Supplementary Information

Supplementary Material 1. Individual effects of conflict and displacement. Spline outputs for the individual effects are in Figures S1 and S2.

Supplementary Material 2. Spline outputs from models. Figures S3 and S4 show the remaining spline outputs for the suspected respiratory infections and suspected diarrheal disease models, respectively.

## Data Availability

Data can be made available from the corresponding author upon reasonable request.
