# Supplementary material for: Climate change, armed conflict, forced displacement, and epidemic-prone diseases: an exploratory study in northern Syria
Source: BMC Public Health. 2025 Aug 4;25:2642. doi: 10.1186/s12889-025-23918-3 (PMC12323065; doi:10.1186/s12889-025-23918-3)
Supplement: Supplementary file 1 — Supplementary Material 1. Individual effects of conflict and displacement. Spline outputs for the individual effects are in Figures S1 and S2. [file 12889_2025_23918_MOESM1_ESM.pdf]

Additional file 1: Individual effects of conflict and displacement

We initially ran the models for suspected respiratory infections and diarrheal disease with conflict events and displacements modeled individually rather than as an interaction term. The spline outputs for suspected respiratory infections can be found in **Figure S1** and for suspected diarrheal disease in **Figure S2**. In both cases, the remaining parametric and non-parametric outputs did not meaningfully change between the two specifications (i.e., modeled individually and as an interaction term). In the suspected respiratory infection model, AIC decreased slightly with the interaction term (98,032.9 versus 98,062.2); the same was true for the suspected diarrheal disease model (90,654.8 versus 90,670.2). Modeling these two variables as an interaction term was also more in line with the contextual realities of northern Syria during the study period, and thus we ultimately chose to include them as an interaction.

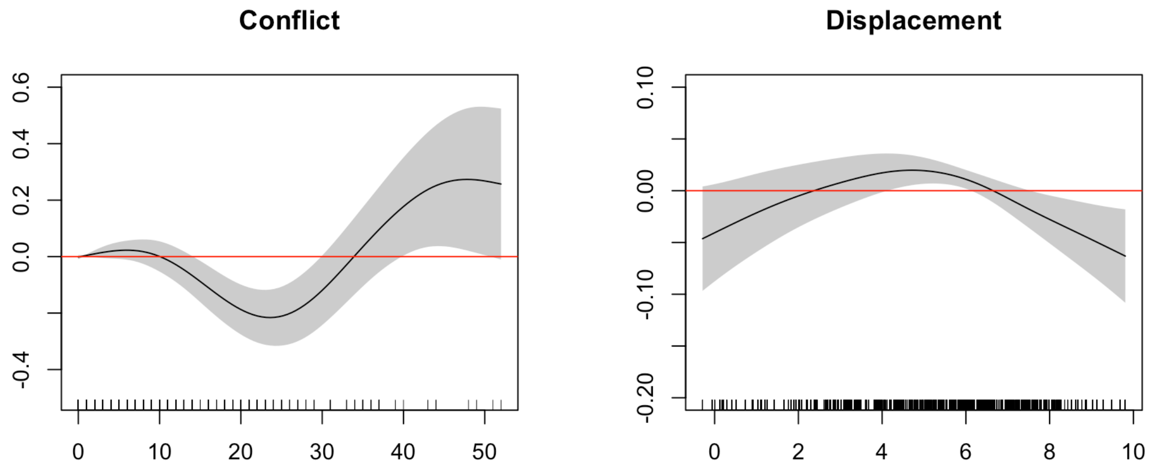

Figure S1: Spline outputs of conflict events and displacement modeled individually in the suspected respiratory infection GAM

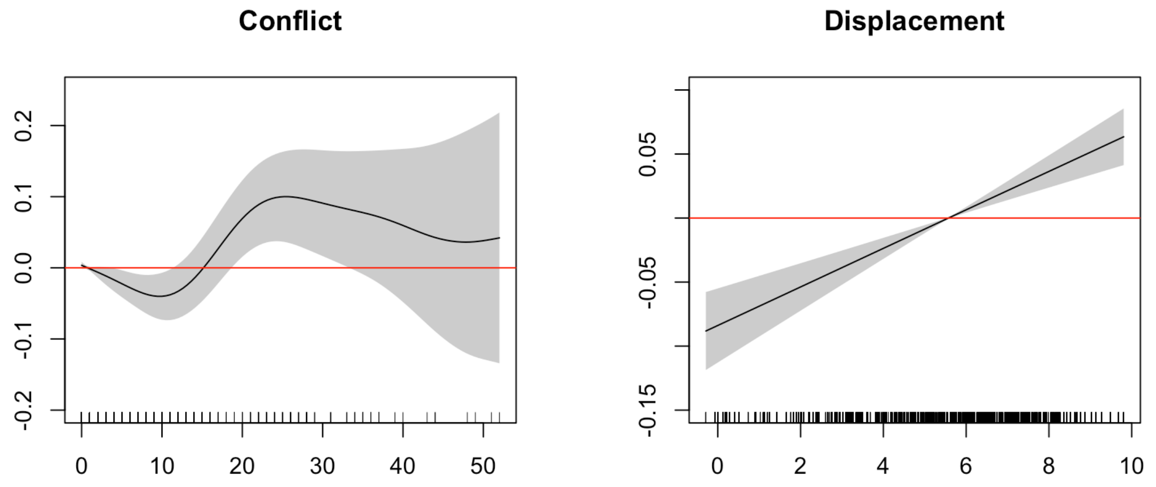

Figure S2: Spline outputs of conflict events and displacement modeled individually in the suspected diarrheal disease GAM
