# Supplementary material for: Climate change, armed conflict, forced displacement, and epidemic-prone diseases: an exploratory study in northern Syria
Source: BMC Public Health. 2025 Aug 4;25:2642. doi: 10.1186/s12889-025-23918-3 (PMC12323065; doi:10.1186/s12889-025-23918-3)
Supplement: Supplementary file 2 — Supplementary Material 2. Spline outputs from models. Figures S3 and S4 show the remaining spline outputs for the suspected respiratory infections and suspected diarrheal disease models, respectively. [file 12889_2025_23918_MOESM2_ESM.pdf]

Additional file 2: Spline outputs from models

The spline outputs from the suspected respiratory infection and diarrheal disease models can be found in **Figure S3** and **S4**, respectively.

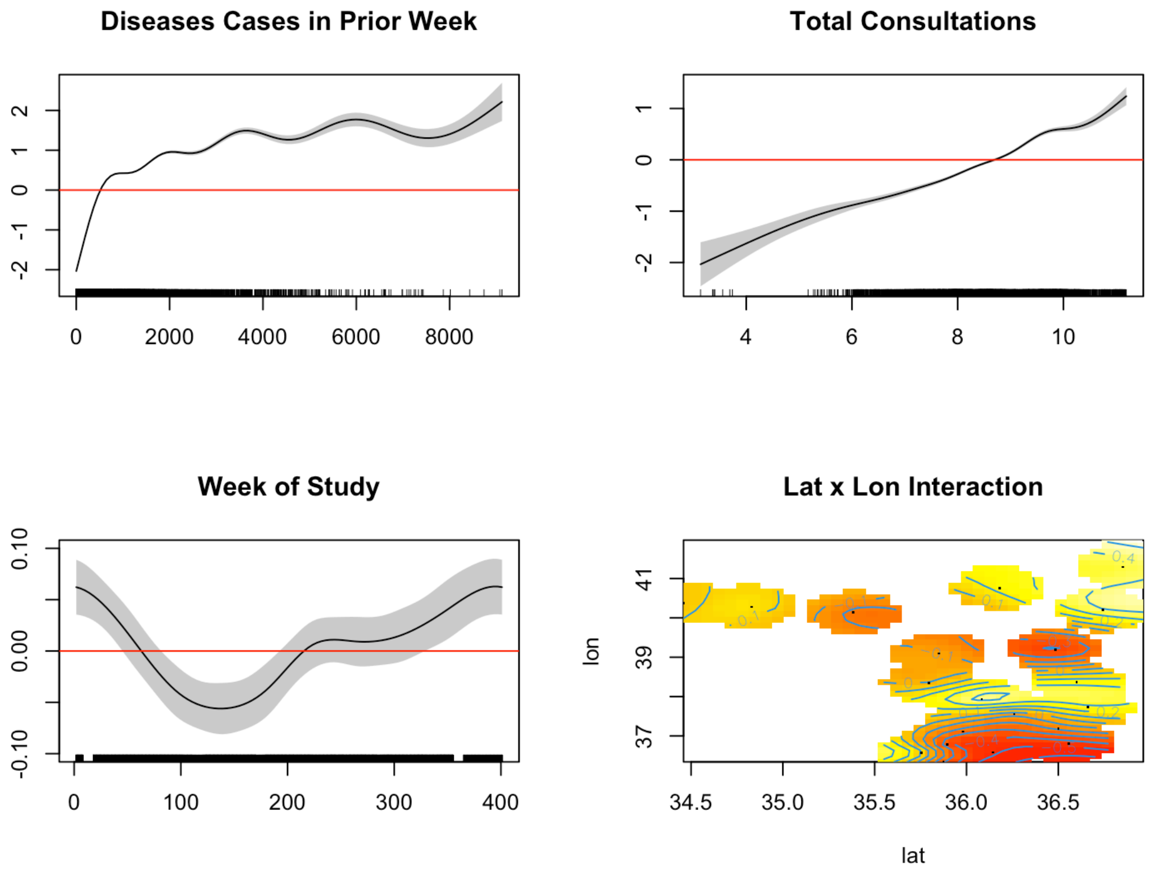

Figure S3: Remaining spline outputs for the suspected respiratory infections model. Outputs for week of year (seasonality) and the conflict and displacement interaction term can be found in **Figure 4**. The splines are considered significant when the black line (IRR) and grey shaded areas (95% CIs) are all either above or below the red line. In the geometric centroids' latitude and longitude interaction, reds indicate lower risk and yellows indicate higher risk.

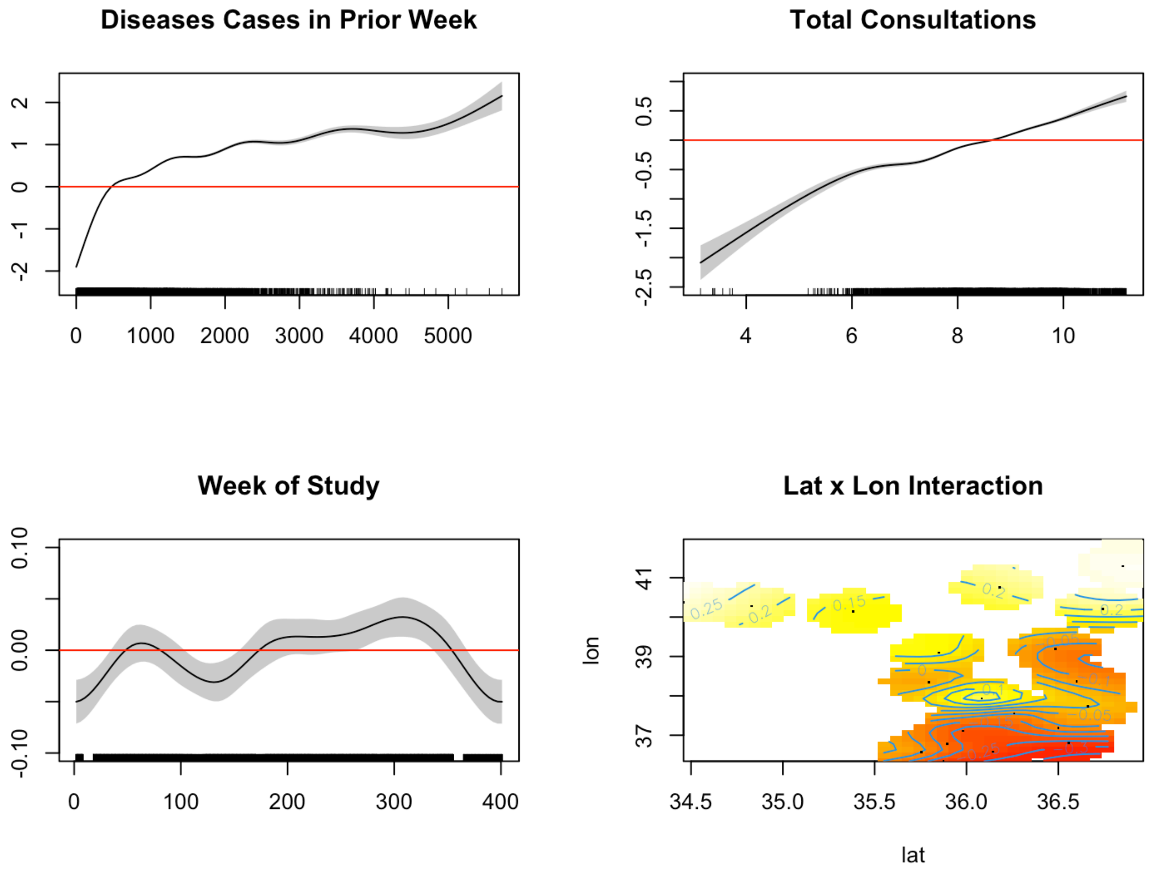

Figure S4: Remaining spline outputs for the suspected diarrheal disease model. Outputs for week of year (seasonality) and the conflict and displacement interaction term can be found in **Figure 4.** The splines are considered significant when the black line (IRR) and grey shaded areas (95% CIs) are all either above or below the red line. In the geometric centroids' latitude and longitude interaction, reds indicate lower risk and yellows indicate higher risk.
